# Role of Pulsed Electromagnetic Field on Alveolar Bone Remodeling during Orthodontic Retention Phase in Rat Models

**DOI:** 10.3390/dj12090287

**Published:** 2024-09-09

**Authors:** Hafiedz Maulana, Yuyun Yueniwati, Nur Permatasari, Hadi Suyono

**Affiliations:** 1 Doctoral Program in Medical Science, Faculty of Medicine, Universitas Brawijaya, Malang 65145, Indonesia; 2 Department of Dentistry-Biomedical Sciences, Oral and Maxillofacial Pathology, Faculty of Dentistry, Universitas Jember, Jember 68121, Indonesia; 3 Department of Radiology, Saiful Anwar General Hospital, Faculty of Medicine, Universitas Brawijaya, Malang 65145, Indonesia; yuyun@ub.ac.id; 4 Department of Pharmacology, Faculty of Medicine, Universitas Brawijaya, Malang 65145, Indonesia; permatasarirazaq.fk@ub.ac.id; 5 Department of Electrical Engineering, Faculty of Engineering, Universitas Brawijaya, Malang 65145, Indonesia; hadis@ub.ac.id

**Keywords:** remodeling, pulsed electromagnetic field, retention phase

## Abstract

Alveolar bone remodeling during the retention phase is essential for successful orthodontic treatment. Pulsed electromagnetic field (PEMF) therapy is an adjunctive therapy for bone-related diseases that induces osteogenesis and prevents bone loss. This study aimed to examine the role of PEMF exposure during the retention phase of orthodontic treatment in alveolar bone remodeling. A total of 36 male Wistar rats were divided into control, PEMF 7, and PEMF 14 groups; a 50 g force nickel–titanium closed-coil spring was inserted to create mesial movement in the first molar for 21 d. Furthermore, the spring was removed, and the interdental space was filled with glass ionomer cement. Concurrently, rats were exposed to a PEMF at 15 Hz with a maximum intensity of 2.0 mT 2 h daily, for 7 and 14 days. Afterwards, the cements were removed and the rats were euthanized on days 1, 3, 7, and 14 to evaluate the expression of Wnt5a mRNA and the levels of RANKL, OPG, ALP, and Runx2 on the tension side. The data were analyzed with ANOVA and post hoc tests, with *p* < 0.05 declared statistically significant. PEMF exposure significantly upregulated Wnt5a mRNA expression, OPG and ALP levels, and Runx2 expression, and decreased RANKL levels in the PEMF 7 and 14 groups compared to the control group (*p* < 0.05). This study showed that PEMF exposure promotes alveolar bone remodeling during the orthodontic retention phase on the tension side by increasing alveolar bone formation and inhibiting resorption.

## 1. Introduction

Alveolar bone remodeling during the retention phase is important for maintaining tooth stability after orthodontic treatment. Alveolar bone remodeling during orthodontic tooth movement begins with bone resorption on the compression side, followed by formation on the tension side [1]. Several key factors are required in this process, including the non-canonical Wnt signaling pathway, runt-related transcription factor 2 (Runx-2), receptor activator of nuclear factor kappa B ligand (RANKL), osteoprotegerin (OPG), and alkaline phosphatase (ALP). Non-canonical Wnt5a signaling can activate canonical Wnt, thereby translocating β-catenin to the cell nucleus and activating osteoblast proliferation [2]. Runx-2 and ALP are important indicators of osteoblast differentiation and bone mineralization [3,4], while RANKL/OPG is a molecular biomarker that plays a role in maintaining homeostasis between bone deposition and resorption [5,6].

The balance of alveolar bone remodeling after orthodontic treatment is a major concern in the success of orthodontic treatment. Although patients have been using retention devices for several years, the tendency of the teeth to return to their initial position (relapse) is inevitable [7]. Therefore, an appropriate method is required to accelerate alveolar bone remodeling during the retention phase of orthodontic treatment. In general, the use of noninvasive methods, such as pulsed electromagnetic field (PEMF), can treat patients with post-menopausal osteoporosis [8,9], accelerate bone formation, and reduce pain in fractures [10,11,12]. The Food and Drug Administration has approved PEMF as a safe and effective treatment for bone repair with no adverse effects [13,14].

Experimental research in cell culture has shown that PEMF exposure increases osteoblast formation and inhibits osteoclast formation [15,16]. According to experiments on rat models of osteoporosis, PEMF stimulates bone regeneration by increasing the remodeling process through the stimulation of osteoblast and osteoclast cell proliferation and function enhancement, such as ALP elevation, RANK/RANKL/OPG regulation, and Wnt signaling [17,18,19,20]. The use of a PEMF device for biostimulation can reduce discomfort and pain due to the pressure generated by orthodontic appliances that accelerate treatment [21,22,23,24].

Based on several previous studies, PEMF is thought to increase bone deposition and promote alveolar bone remodeling. This is the first study to evaluate the effects of PEMF exposure on the retention phase of orthodontics. Moreover, it aimed to explore the effects of exposure to PEMF during the retention phase of orthodontics on alveolar bone remodeling in a rat model.

## 2. Materials and Methods

### 2.1. Experimental Animal Models

All phases of the research were approved by the Ethics Committee of the Faculty of Medicine, Brawijaya University (number 218/EC/KEPK-S 3/09/2022). The number of samples was 36 male Wistar rats (*Rattus norvegicus*) aged 12 weeks, of 250–300 g weight. Rats were housed in separate cages with a constant temperature of around 23 °C and received adequate nutrition and liquids each day. The animal was assessed daily, while body weight was monitored fortnightly, starting one week before the experiment. Furthermore, weight loss of 15% during the experimental period was an exclusion criterion.

The experimental animals were divided into (1) the control group, fitted with an orthodontic device for 21 days, with a retention phase lasting for 7 days, and no PEMF stimulation; (2) the PEMF 7 group, fitted with an orthodontic device for 21 days, and with a retention phase with PEMF stimulation for 7 days; and (3) the PEMF 14 group, fitted with an orthodontic device for 21 days, and with a retention phase with PEMF stimulation for 14 days. The anesthesia procedure was performed using an intramuscular injection of a 1 kg/mL dose of ketamine hydrochloride and xylazine in a 1:1 ratio.

### 2.2. Orthodontic Tooth Movement in Rat Models

**Figure 1:**
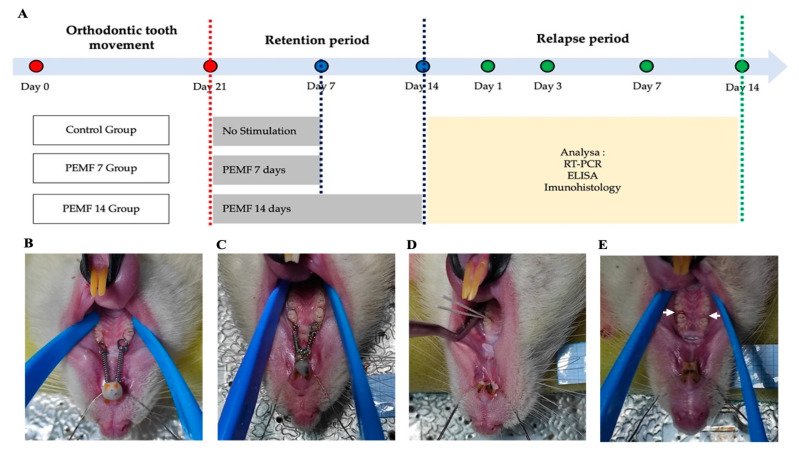
Research design. (**A**) PEMF stimulation phases, (**B**) orthodontic appliance installation, (**C**) post orthodontic tooth movement, (**D**) absorption of GCF sample with paper points, and (**E**) sampling region (white arrow) for RT-PCR. PEMF: pulsed electromagnetic field, GCF: gingival crevicular fluid, RT-PCR: real-time polymerase chain reaction.

Figure 1A shows the stages of the research. An orthodontic device in the form of a nickel–titanium closed-coil spring (American Orthodonti, Sheboygan, WI, USA) with a diameter of 0.01 inch and a length of 5 mm was inserted between the maxillary molar 1 and incisor teeth, as shown in Figure 1B. The force produced by the orthodontic device was 50 gF (gram force) to shift the 1st molar tooth mesially and bodily [18], and the tooth movement lasted for 21 days (Figure 1C).

### 2.3. Retention Phase

The retention phase started after 21 days, and the active orthodontic movement process was completed [25]. The interdental space between the first and second molars was cleaned and dried. The interdental space was filled evenly and parallel to the occlusal surface using Fuji type II light-cured glass ionomer cement (GC, Tokyo, Japan) [26]. In addition, the nickel–titanium closed-coil spring was removed after the interdental space was filled with a glass ionomer to prevent immediate relapse.

### 2.4. PEMF Exposure

**Figure 2:**
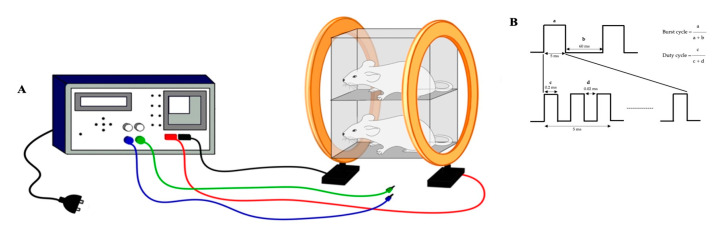
PEMF stimulator. (**A**) The PEMF device and rats were kept in a special fiber cage, placed between a Helmholtz coil and exposed 2 h/day. (**B**) The waveform was square with a burst width of 5 ms, burst wait of 60 ms, pulse width of 0.2 ms, pulse wait of 0.02 ms, pulse rise of 0.3 μs, and pulse fall of 2.0 μs.

The PEMF device was assembled and calibrated based on previous research [27,28,29,30], as shown in Figure 2. The waveform was square with a burst width of 5 ms, burst wait of 60 ms, pulse width of 0.2 ms, pulse wait of 0.02 ms, pulse rise of 0.3 μs, and pulse fall of 2.0 μs. The maximum magnetic field intensity generated was 2.0 mT, with a repetition frequency of 15 Hz [28,29]. To assess the effect of PEMF exposure on the retention phase after orthodontic tooth movement, rats were kept in a special fiber cage and placed on a stimulator rack and stimulated for 2 h/day for 7 or 14 days.

### 2.5. Enzyme-Linked Immunosorbent Assay (ELISA) Analysis

After completing the retention phase for 7 and 14 days, the glass ionomer cement in the interdental space was removed. The rats were euthanized on days 1, 3, 7, and 14 for further analysis. After the rats were anesthetized, their extremities were immobilized on the operating board, and the oral cavity was kept open using a brace. The regions around the first and second molars were cleaned and dried using a cotton roller. A sterile number 15 paper point was inserted into the gingival sulcus at the distal part of molar 1 and we waited for 60 s for gingival crevicular fluid (GCF) sample collection (Figure 1D). Subsequently, the paper point was withdrawn and inserted into a micro-centrifuge tube containing 350 μL phosphate-buffer saline. In this context, the GCF samples collected were centrifuged at 1000× *g* and 4 °C for 20 min. The supernatants were put in a tube and stored at −80 °C. The levels of RANKL, OPG, and ALP were analyzed with ELISA methods (Rat RANKL: E-EL-R0841, Rat OPG: E-EL-R3005, and Rat ALPL: E-EL-R1109 ELISA Kit Elabscience, Houston, TX, USA).

### 2.6. Reverse Transcription Polymerase Chain Reaction (RT-PCR) Analysis

The sample for RT-PCR analysis was tissues located distal to the first molar (tension side), containing the gingiva, alveolar bone, and periodontal ligament, as shown in Figure 1E. The sample size was 2 mm × 2 mm and was cut using surgical instruments and a low-speed diamond disk. The samples were placed in a microcentrifuge tube and stored in a refrigerator at −80 °C. Pulverization was performed using a mortar and pestle until the ribonucleic acid (RNA) was homogeneously extracted. Total RNA was isolated using an RNAsimple Total RNA Kit (4992858, TIANGEN, Beijing, China). The obtained RNA was reverse-transcribed with a FastKing RT Kit (with gDNase) (Cat. no. 4992223/4992224/4992250; TIANGEN, Beijing, China), using an HRM Analysis Kit (EvaGreen) (Cat. no. 4992776/4992873, TIANGEN, Beijing, China). Primers for Wnt5a and Beta-actin were determined using the NCBI BLAST Server. Table 1 lists the primer sequences, melting temperatures, product sizes, and GenBank accession numbers. RNA expression levels were measured as the ratio of each gene to Beta-actin using semi-quantitative analysis.

RT-PCR was performed using a CFX Opus 96 Real-Time PCR System (Bio-Rad, Hercules, CA, USA). RT-PCR analysis lasted 40 cycles, with denaturation, annealing, melting curve, and extension temperatures of 95 °C for 120 s, 95 °C for 10 s, 60 °C for 20 s, and 72 °C for 30 s, respectively. Beta-actin was used as an endogenous control, and experiments were performed in parallel under the same conditions.

### 2.7. Immunohistochemistry Procedures

After fixation and decalcification, the tissue samples were cut with a thickness of 3 microns. After deparaffinization, the slides were incubated with the primary antibody Runx2 (27-K) (sc-101145, Santa Cruz, Dallas, TX, USA) at 4 °C overnight. The samples were incubated with a secondary antibody-conjugated enzyme-labeled polymer (N-Histofine High Stain HRP, Nichirei Biosciences Inc., Tokyo, Japan), chromogenic substrate, and Mayer hematoxylin solution, according to the manufacturer’s protocols. Runx2-positive cells were characterized by the number of brown osteoblasts, which were calculated using an Optilab camera and Image G ver. 4.0 software on the tension sides with light microscopy at 400× magnification in three selected captured pictures, and calculated as an average in the alveolar bone.

### 2.8. Statistical Analysis

One-way ANOVA parametric and post hoc tests were conducted using SPSS software (version 26.0; SPSS Inc., Chicago, IL, USA). Meanwhile, a *p* < 0.05 was declared statistically significant.

## 3. Results

### 3.1. Wnt5a mRNA Expressions

**Figure 3:**
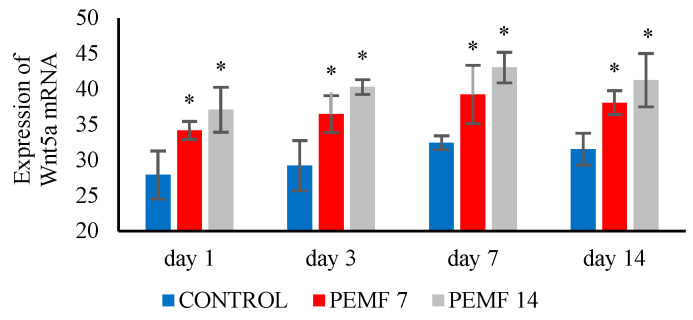
The histogram of Wnt5a mRNA expression. *: *p* < 0.05, significant compared with control group. PEMF: pulsed electromagnetic field.

The Wnt5a mRNA expression was estimated using RT-PCR. Figure 3 shows that the mRNA expression of Wnt5a in the PEMF 7 and 14 groups was significantly elevated (*p* < 0.05) compared to that in the control group.

### 3.2. RANKL, OPG, and ALP Levels

**Figure 4:**
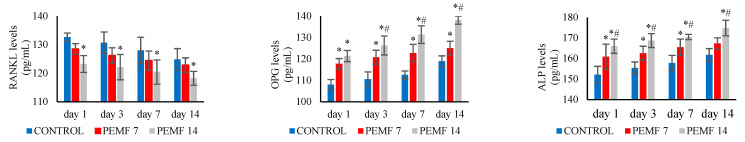
The histogram of RANKL, OPG, and ALP levels. *: *p* < 0.05, significant compared with control group; #: *p* < 0.05, significant compared with PEMF 7 group. PEMF: pulsed electromagnetic field, RANKL: receptor activator of nuclear factor-kappa B ligand, OPG: osteoprotegerin, ALP: alkaline phosphatase.

According to the ELISA results, there was a decrease in the level of RANKL in the PEMF 7 and 14 groups compared with that in the control group, as shown in Figure 4. Significant differences were observed between the 14-day PEMF and control groups (*p* < 0.05). Meanwhile, the levels of OPG and ALP increased in the PEMF 7 and 14 groups compared to those in the control group. Significant differences were observed between the 14-day and 7-day PEMF groups and the control group (*p* < 0.05).

### 3.3. Runx2 Expressions

**Figure 5:**
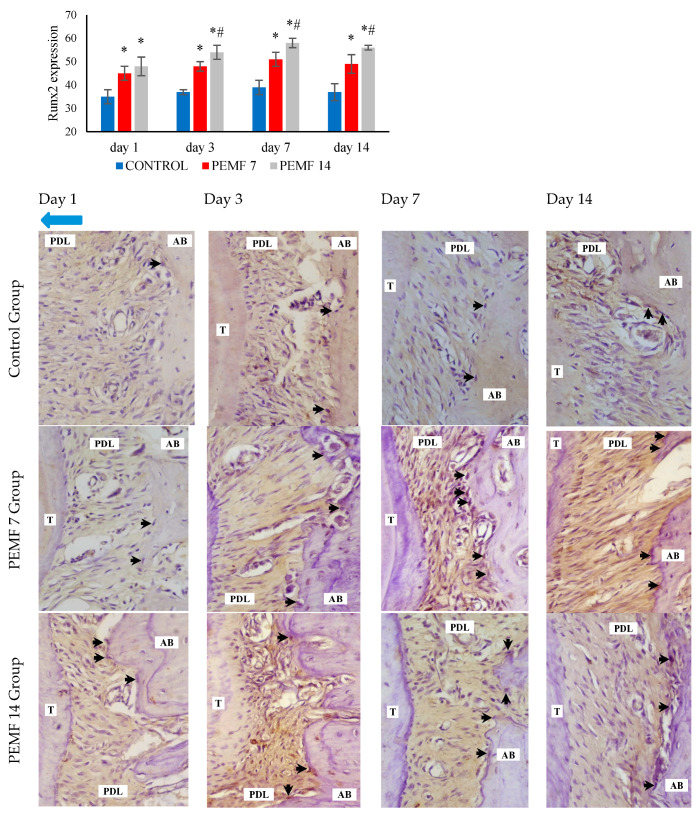
Histogram and immunohistochemical image of Runx2 expression. Runx2 positive-osteoblast (black arrow) and the direction of tooth movement (blue arrow). *: *p* < 0.05, significant compared with control group; #: *p* < 0.05, significant compared with PEMF 7 group. PEMF: pulsed electromagnetic field, T: tooth, PDL: periodontal ligament, AB: alveolar bone, Runx2: runt-related transcription factor 2.

Immunohistochemical analysis showed an increase in Runx2 expression in the PEMF 7 and PEMF 14 groups compared to that in the control group on the tension side, as shown in Figure 5. Significant differences were observed between the 14-day and 7-day PEMF groups and the control group (*p* < 0.05). Runx2 expression in osteoblasts, determined using immunohistochemical analysis of the tension sides, is shown in Figure 5.

## 4. Discussion

The retention phase is the final stage of orthodontic treatment, which aims to prevent relapse and maintain the correct position of teeth after active orthodontic movement [31]. The causes of relapse are multifactorial, but the main factor is incomplete periodontal tissue remodeling. Thus, alveolar bone remodeling is a determining factor in maintaining tooth stability after orthodontic treatment. The results of this research indicate that PEMF exposure in the retention phase for 7 and 14 days increases bone formation and inhibits alveolar bone loss on the areas of tension in rat models after orthodontic tooth movement. Previous studies have presented that PEMF stimulation during implant treatment can effectively increase the density of alveolar bone around the implant, improving osteointegration and implant stability in patients [32].

To uncover the mechanism by which PEMF stimulates bone remodeling after orthodontic tooth movement, we assessed the non-canonical signaling pathway Wnt5a. The results exhibited that Wnt5a mRNA expression on the tension side of the PEMF 7 and 14 groups was upregulated. Wnt5a is a signaling pathway that regulates the process by which bone marrow mesenchymal stromal cells (BMSCs) become mature osteoblasts, suppress osteoblast apoptosis, and promote osteoblast proliferation [33]. Wnt5a upregulates low-density lipoprotein receptor-related protein 5 (Lrp5) and low-density lipoprotein receptor-related protein 6 (Lrp6), which trigger osteoblast differentiation [34]. Wnt5a plays a role in elevating alveolar bone and periodontal ligament remodeling on the areas of tension after tooth movement in a mouse model [35]. A study on PEMF exposure showed increased bone healing, characterized by an increase in Wnt5a mRNA expression [36].

In this study, PEMF exposure for 7 and 14 d upregulated Runx2 expression on the tension side. Runt-related transcription factor 2 (Runx2) regulates the differentiation in mesenchymal cells into osteoblasts and is an early marker of osteogenesis [37]. Li et al. described that PEMF exposure in rats with type 2 diabetes led to improved bone quality characterized by increased Runx2 expression [19]. In rat models of distraction osteogenesis, exposure resulted in the promotion of new bone formation, which was characterized by a significant increase in Runx2 gene expression [38]. A study in a mouse model of osteoporosis investigating the effects of four weeks of PEMF exposure reported that PEMF reduced bone loss in mice and promoted osteogenic differentiation, as indicated by increased Runx-2 expression [39].

After 7 and 14 d of PEMF exposure, ALP and OPG protein levels significantly increased, whereas RANKL levels decreased. ALP is an osteoblast differentiation biomarker used in the early stages of osteoblast differentiation, proliferation, and migration. RANKL/OPG signaling is involved in osteoclast differentiation and activity. These results are consistent with previous analyses, which reported that PEMF exposure increases ALP protein levels [15,29,38,39,40,41,42]. RANKL is a cell surface protein that binds to RANK on osteoclast membranes to stimulate differentiation. Similarly, OPG inhibited osteoclast maturation and differentiation [43,44,45]. PEMF can exert a suppressive effect on osteoclastogenesis through the RANK/RANKL/OPG signaling pathway. Intraperitoneal administration of an anti-mRANKL Ab in rat models of orthodontic tooth movement decreased the tartrate-resistant acid phosphatase (TRAP) expressions [46]. These data are supported by Zhou et al., who showed that PEMF stimulation in osteoporosis model rats resulted in a decrease in RANKL and an elevation in OPG [47]. Catalano et al. also reported that stimulation of postmenopausal women resulted in a decrease in the RANKL/OPG ratio [9].

In this study, tooth movement in experimental animals lasted for 21 days. Furthermore, the retention stage was extended to 7 and 14 d. Previous research on the observation of alveolar bone structure in adolescent rats with molar tooth movement for 14 days showed bone deposition, which was marked by an increase in alveolar bone density and trabecular thickness in the tension and compression areas [48]. Therefore, orthodontic tooth movement for 21 days in the rat model could be considered complete. Biomarkers of alveolar bone remodeling in the PEMF 14 group showed an increase in Wnt5a mRNA, ALP, and OPG, and a decrease in RANKL compared to the PEMF 7 group. These results are in accordance with those of previous studies, wherein the retention phase for two weeks reduced orthodontic relapse in experimental animals [26].

The results of this study will contribute positively to the application of PEMF therapy in patients undergoing orthodontic treatment. The effect of PEMF stimulation on the acceleration of alveolar bone remodeling in the retention phase provides a new treatment strategy that supports the outcomes of orthodontic treatment in clinical practice. However, this study has some limitations because it only provided gene and protein data. Further investigations using histomorphometry and microCT analysis are needed to determine the structure of the alveolar bone and periodontal tissue post-orthodontic tooth movement. In addition, the application of this treatment requires further research to study the effects of PEMF exposure, both locally and systemically, in clinical trials. Thus, PEMF is expected to be an effective additional procedure for accelerating orthodontic treatment.

## 5. Conclusions

This study demonstrated that PEMF exposure during the retention phase accelerates alveolar bone remodeling after orthodontic tooth movement on the tension side. Exposure to a PEMF for 14 days effectively promoted alveolar bone deposition and inhibits alveolar bone resorption mediated by Wnt5a signaling.

## Figures and Tables

**Table 1:** Primer sequence for RT-PCR analysis.

Gen Target	Primer	Primer Sequence (5′–3′)	Product Length (bp)	Tm (°C)	Gene Bank
Wnt5a	Forward	CGCTGCTGGAGTGGTAAATGC	148	59.90	NM_022631.3
	Reverse	TGCGACCTGCTTCATTGTTG		60.04	
Beta-actin	Forward	CCTAAGGCCAACCGTGAAA	152	55.3	NM_017008.4
	Reverse	CAGAGGCATACAGGGACAAC		55.2	

## Data Availability

The original contributions presented in the study are included in the article; further inquiries can be directed to the corresponding author.
